# Eradication of Biofilms on Catheters: Potentials of *Tamarix ericoides* Rottl. Bark Coating in Preventing Catheter-Associated Urinary Tract Infections (CAUTIs)

**DOI:** 10.3390/life14121593

**Published:** 2024-12-03

**Authors:** Mohammed H. Karrar Alsharif, Muhammad Musthafa Poyil, Salman Bin Dayel, Mohammed Saad Alqahtani, Ahmed Abdullah Albadrani, Zainab Mohammed M. Omar, Abdullah MR. Arafah, Tarig Gasim Mohamed Alarabi, Reda M. Fayyad, Abd El-Lateef Saeed Abd El-Lateef

**Affiliations:** 1 Department of Basic Medical Sciences, College of Medicine, Prince Sattam bin Abdulaziz University, Al-Kharj 11942, Saudi Arabia; m.poyil@psau.edu.sa (M.M.P.); z.omar@psau.edu.sa (Z.M.M.O.); a.arafah@psau.edu.sa (A.M.A.); as.ismail@psau.edu.sa (A.E.-L.S.A.E.-L.); 2 Department of Internal Medicine, College of Medicine, Prince Sattam bin Abdulaziz University, Al-Kharj 11942, Saudi Arabia; s.bindayel@psau.edu.sa (S.B.D.); ms.alqahtani@psau.edu.sa (M.S.A.); a.albadrani@psau.edu.sa (A.A.A.); 3 Department of Pharmacology, Faculty of Medicine, Al-Azhar University, Assiut 71524, Egypt; 4 Department of Anatomy, College of Medicine, King Khalid University, Abha 61421, Saudi Arabia; tarigfuture@gmail.com; 5 Department Pharmacology, General Medicine Practice Program, Batterjee Medical College, Asser 61961, Saudi Arabia; drredafayyadccmorc@gmail.com; 6 Department of Pharmacology, Faculty of Medicine, Al-Azhar University, Cairo 11511, Egypt

**Keywords:** biofilms, catheter coatings, catheter-associated urinary tract infections (CAUTIs), confocal microscopy, GC-MS, *Tamarix ericoides* Rottl. bark

## Abstract

Catheter-associated urinary tract infections (CAUTIs) cause serious complications among hospitalized patients due to biofilm-forming microorganisms which make treatment ineffective by forming antibiotic-resistant strains. As most CAUTI-causing bacterial pathogens have already developed multidrug resistance, there is an urgent need for alternative antibacterial agents to prevent biofilms on catheter surfaces. As a trial to find out such a potential agent of natural origin, the bark of *Tamarix ericoides* Rottl., a little-known plant from the Tamaricaceae family, was examined for its antibacterial and antibiofilm activities against one of the major, virulent, CAUTI-causing bacterial pathogens: *Enterococcus faecalis*. The methanolic *T. ericoides* bark extract was analyzed for its antibacterial activity using the well diffusion method and microdilution method. Killing kinetics were calculated using time–kill assay, and the ability of biofilm formation and its eradication upon treatment with the *T. ericoides* bark extract was studied by crystal violet assay. GC-MS analysis was performed to understand the phytochemical presence in the extract. A in vitro bladder model study was performed using extract-coated catheters against *E. faecalis*, and the effect was visualized using CLSM. The changes in the cell morphology of the bacterium after treatment with the *T. ericoides* bark extract were observed using SEM. The biocompatibility of the extract towards L_929_ cells was studied by MTT assay. The anti-*E. faecalis* activity of the extract-coated catheter tube was quantified by viable cell count method, which exposed 20% of growth after five days of contact with *E. faecalis*. The anti-adhesive property of the *T. ericoides* bark extract was studied using CLSM. The extract showed potential antibacterial activity, and the lowest inhibitory concentration needed to inhibit the growth of *E. faecalis* was found to be 2 mg/mL. The GC-MS analysis of the methanolic fractions of the *T. ericoides* bark extract revealed the presence of major phytochemicals, such as diethyl phthalate, pentadecanoic acid, methyl 6,11-octadecadienoate, cyclopropaneoctanoic acid, 2-[(2-pentylcyclopropyl) methyl]-, methyl ester, erythro-7,8-bromochlorodisparlure, etc., that could be responsible for the antibacterial activity against *E. faecalis.* The killing kinetics of the extract against *E. faecalis* was calculated and the extract showed promising antibiofilm activity on polystyrene surfaces. The *T. ericoides* bark extract effectively reduced the *E. faecalis* mature biofilms by 75%, 82%, and 83% after treatment with 1X MIC (2 mg/mL), 2X MIC (4 mg/mL), and 3X MIC (6 mg/mL) concentrations, respectively, which was further confirmed by SEM analysis. The anti-adhesive property of the *T. ericoides* bark extract studied using CLSM revealed a reduction in the biofilm thickness, and the FDA and PI combination revealed the death of 80% of the cells on the extract-coated catheter tube. In addition, SEM analysis showed extensive damage to the *E. faecalis* cells after the *T. ericoides* bark extract treatment, and it was not cytotoxic. Hence, after further studies, *T. ericoides* bark extract with potential antibacterial, antibiofilm, and anti-adhesive activities can be developed as an alternative agent for treating CAUTIs.

## 1. Introduction

Major nosocomial infections in the medical field are mainly associated with medical devices that provide healthcare support for hospitalized patients. The longer time usage of lifesaving medical devices like indwelling catheters, prosthetic joints, ventilators, etc., due to various reasons, create unpredictable complications when the devices are not handled properly, resulting in slight to severe medical device-associated infections which have been well documented [1,2,3]. Among them, catheter-associated urinary tract infection (CAUTI) is one of the most significant nosocomial infections due to the abundant catheter usage in hospitalized patients for recovery from it [4,5,6,7]. Even though the urinary tract is generally sterile, when a small urine blockage or the use of an indwelling catheter happens, they provide entry for uropathogens from the outer environment through the catheter lumen which leads to the formation of microbial colonies on the catheter surfaces, resulting in mild to severe and complicated catheter-associated urinary tract infections (CAUTIs) which affect more than several million people globally [8,9,10]. Though various causative agents are involved in CAUTIs, *Enterococcus faecalis* is one of the most important Gram-positive organisms that form biofilms on living and non-living surfaces [11,12,13,14]. Even though Ampicillin is considered a drug of choice against infections including CAUTIs caused by *E. faecalis* [15,16], most of the time the treatment will be very difficult as the bacterium has many unique pathogenic characteristics and virulence factors, including its ability to establish urinary tract infections by overcoming foreign body-mediated inflammation, its host fibrinogen-dependent biofilm formation ability using Ebp pilus, its ability to subvert or evade immune-mediated clearance, etc. [17,18,19,20]. Biofilm formation is a technique of *E. faecalis* to escape from antibiotic treatment and is performed by forming a complex three-dimensional slimy structure that consists of extracellular polymeric substances that make the treatment and management of the infection complicated, leading to elevated morbidity and mortality rates [21,22]. In CAUTIs, when antibiotic treatment is practiced for a longer time, it may lead to antibiotic-resistant strain development by several mechanisms, resulting in CAUTIs’ treatment failure [23,24,25]. For CAUTI prevention, coating catheters with antibacterial agents like silver, antibiotics, and anti-adhesive materials such as polytetrafluoroethylene, hydrogels [26,27], etc., is in practice. Though these techniques have been used, many reports say that these coated catheters are not able to reduce symptomatic CAUTIs significantly when compared to standard catheters [28,29]. Also, the use of drug coating may lead to an antibiotic resistance increase [30]. Therefore, scientists are looking for novel coating agents for catheter surfaces to prevent biofilm formation.

Over several decades, medicinal plants have been extensively used in treating human diseases, owing to their secondary metabolites such as phenols, flavonoids, etc. [31,32,33]. The WHO says that one-third of the world’s population uses traditional plant-based folk medicine for their primary healthcare treatment [34]. Scientists are searching back in the plant world for novel antibiotic compounds as bacterial pathogens find it difficult to develop resistance towards plant extracts because of many reasons including multiple targeting and synergic activities by multiple bioactive compounds [35,36,37,38]. *Tamarix ericoides* Rottl. is a ‘not well-known’ plant from the Tamaricaceae family, which has been reported to have the capacity to cure gastrointestinal disorders, wounds, dental problems, diabetes, etc., and is also known to possess anti-inflammatory properties [39,40]. So, in a trial to search for new bioactive compounds with novel modes of action against one of the most dreaded, CAUTI-causing bacterial pathogens, the present study investigated the antibacterial, antibiofilm, and anti-adhesive properties of a methanolic extract of *T. ericoides* bark against *E. faecalis*.

## 2. Materials and Methods

### 2.1. Chemicals and Preparation of Inoculum

The fluorescein diacetate (FDA) and propidium iodide (PI) used in this study were purchased from Sigma Aldrich, Louis, MO, USA. Brain–heart infusion agar (BHIA) and the positive control, Ampicillin, were bought from Hi Media, India. The cultured *Enterococcus faecalis* (ATCC 29212), procured from the American Type Culture Collection, was used for preparing a 0.5 MacFarland unit overnight culture (10^6^ CFU/mL) for the entire study. Methanol was used as the vehicle control.

### 2.2. Preparation of T. ericoides Bark Methanolic Extract

The coarse powder obtained after grinding the sun-dried bark of the collected medicinal plant *T. ericoides* was used to fill in the cellulose thimble of the Soxhlet apparatus, and this was followed by methanol addition, as per the standard protocol [41]. The reaction was continued at 60 °C for several hours until a clear methanol solution was obtained. The crude methanolic extract was obtained after solvent evaporation.

### 2.3. GC-MS Profiling of T. ericoides Bark Methanolic Extract

The chemical profiling of the bioactive fractions present in the methanolic extract of the *T. ericoides* bark was performed using gas chromatography and mass spectroscopy (GC-MS) as per standard procedures [42]. Before the start of the experiment, the necessary settings such as the temperature, gas, etc., were initiated and the methanolic sample (1 µL) was injected using a micro-syringe, and this was followed by continuous scanning for 30 min. The eluted compounds were separated from the column and were detected. The individual compounds present in the methanolic fractions were represented as each peak in a chromatogram and were entered into a mass spectroscopy detector. Later, the identification of the separated compounds was completed by correlating the mass spectra patterns and retention indices present in the computer library.

### 2.4. Antibacterial Activity of T. ericoides Bark Methanolic Extract

The antibacterial activity of the *T. ericoides* bark methanolic extract was examined against the selected bacterial pathogen *E. faecalis* through the well diffusion method [43]. In brief, sterile BHIA plates were swabbed with *E. faecalis* overnight cultures, and wells were made on the plates. The wells were added with various concentrations of *T. ericoides* bark methanolic extract, and the diameters of the growth inhibition zones after the incubation represented the antibacterial bacterial activity of the extract against the *E. faecalis*.

### 2.5. Determination of MIC of T. ericoides Bark Methanolic Extract

The minimum inhibitory concentration of the *T. ericoides* bark methanolic extract against *E. faecalis* was determined by the microdilution plate method [44]. Briefly, 4 mg/mL of *T. ericoides* bark extract was diluted serially in a BHI broth until the final concentration reached 0.03 mg/mL following an *E. faecalis* overnight culture addition, and the mixture was incubated. Later, each well was measured at 600 nm for turbidity using an Elisa reader.

### 2.6. Time–Kill Effect of T. ericoides Bark Extract

The killing ability of the *T. ericoides* bark extract on *E. faecalis* was calculated using time–kill assay [44]. In short, 2 mg/mL of *T. ericoides* bark extract was added to 1 × 10^6^ CFU/mL of an *E. faecalis* overnight culture and incubated for various time intervals such as 0 h, 1 h, 2 h, 4 h, 6 h, and 12 h, followed by sample collection at each time point. The collected treated and untreated samples underwent 10-fold serial dilution, and spread plating was conducted for each dilution to observe the viable cell count.

### 2.7. Effect of T. ericoides Bark Extract on E. faecalis Biofilm Formation

To understand the effect of the *T. ericoides* bark extract ability on *E. faecalis* biofilm formation, crystal violet assay [44] was performed. In brief, various concentrations of *T. ericoides* bark extract ranging from 4 mg/mL to 0.03 mg/mL were added to 96-well plates that contained an *E. faecalis* overnight culture and were added with BHI broth, and the mixtures were left for 5 days. Later, the phosphate buffer saline (PBS)-washed wells were subjected to methanol fixation, followed by staining with crystal violet. The destaining was conducted by adding an acetone–ethanol complex, and the percentage of biofilm formation was calculated after the obtained product was read at 570 nm.

### 2.8. Effect T. ericoides Bark Extract on Eradication of Biofilms by E. faecalis

To understand the result of the *T. ericoides* bark extract treatment on the mature *E. faecalis* biofilms, qualitative and quantitative biofilm assays were performed [44]. In the qualitative assay, an overnight culture of *E. faecalis* was grown for 5 days on Whatman No.1 filter paper and was treated with *T. ericoides* bark extract at a concentration of 2 mg/mL for 1 h, followed by a PBS wash. The strips containing the attached cells were left for glutaraldehyde fixation and subsequently subjected to ethanol dehydration. The air-dried treated and untreated strips coated with gold were analyzed for biofilm eradication using SEM (Supra 55, Carl Zeiss, Wetzlar, Germany). On the other hand, the quantitative *E. faecalis* biofilm eradication was studied after treatment with *T. ericoides* bark extract through crystal violet assay [44]. The assay was conducted on polystyrene surfaces on which the *E. faecalis* overnight culture was allowed to form biofilm for 5 days. Then, it was subjected to PBS washing, and the mature biofilms were allowed to react with three various concentrations of *T. ericoides* bark extract, 2 mg/mL (1X MIC), 4 mg/mL (2X MIC), and 6 mg/mL (3X MIC), for 24 h. Then, the treated and untreated mature biofilms were fixed with methanol, and this was followed by staining with crystal violet. The destained final products were measured for their optical densities at 570 nm to calculate the percentage of biofilm eradication after the treatment.

### 2.9. T. ericoides Extract-Coated In Vitro Bladder Model

The anti-*E. faecalis* effect of the *T. ericoides* bark extract-coated catheter was studied using an in vitro bladder model [45]. A sterile silicon catheter cutting coated with *T. ericoides* bark extract was air-dried and placed over an *E. faecalis* overnight culture lawn on a BHA plate. A clear growth inhibition zone formation around the catheter tube indicated the antibacterial activity of the *T. ericoides* bark extract against the *E. faecalis*.

### 2.10. Quantification of Bacterial Load on In Vitro Bladder Model

The quantification of *E. faecalis* bacteria on catheters coated with *T. ericoides* bark extract was conducted using the colony count method as per standard procedures [46]. In brief, the uncoated and the extract-coated catheter tubes were immersed in an overnight *E. faecalis* culture containing BHI broth for 24 h, and then the attached cells from both catheter tubes were collected by vigorous shaking. Later, the viable cells were counted by carrying out serial dilution (10-fold) of the coated as well as uncoated catheter tubes. On the other hand, the turbidity was measured at 600 nm to calculate the growth percentage for the coated and uncoated catheter tubes.

### 2.11. Visualization of Biofilms on Bladder Model

The anti-adhesive properties of the *T. ericoides* bark extract on the catheter surface against *E. faecalis* were explored using confocal laser scanning microscopy (CLSM) as per standard procedures [46]. For the analysis, *T. ericoides* bark extract-coated and uncoated catheter tubes were left for *E. faecalis* biofilm formation for up to 5 days and then the washed catheter tubes were subjected to fluorescein diacetate (FDA, 40 µL from 5 mg/mL) staining for 10 min, followed by propidium iodide (PI, 20 µL from 1 mg/mL) staining for 5 min. Then, the biofilms were observed on both tubes using CLSM, and the live/dead cell percentages were calculated.

### 2.12. Effect of T. ericoides Bark Extract on E. faecalis Cell Morphology

The effect of the *T. ericoides* bark extract on *E. faecalis* cell morphology was studied using scanning electron microscopy analysis [47]. For the analysis, *E. faecalis* was allowed to grow on Whatmann No.1 filter paper strips followed by *T. ericoides* bark extract (2 mg/mL) treatment for 1 h. Then, glutaraldehyde fixation for washed strips was performed, followed by dehydration with an ethanol gradient. Gold coating was performed for both the treated and untreated strips, and the images were observed for morphological changes after the treatment using SEM (Supra 55, Carl Zeiss).

### 2.13. Cytotoxicity of T. ericoides Bark Extract

The effect of the *T. ericoides* bark extract on the L_929_ (mouse fibroblast) cell line was studied using MTT assay [44]. In short, different concentrations (1, 2, 3, 4, and 5 mg/mL) of *T. ericoides* bark extract were added to the wells containing L_929_ cells grown in Dulbecco’s Modified Eagle’s Medium (DMEM) with 10% fetal bovine serum and incubated. Then, the development of formazan formation was achieved when the MTT solution was added, and it was dissolved using DMSO. The optical density of the purple-colored product was read at 570 nm to calculate the cell viability percentage after the treatment.

### 2.14. Statistical Analysis

For the determination of the minimal inhibitory concentrations (MICs), determination of the effect of the extract on biofilm formation, and biofilm eradication, the mean and the standard deviations were calculated. Also, for the quantification of the bacterial load and live/dead assay, the statistical significance testing was carried out through Student’s *t*-tests. A *p*-value ≤ 0.05 was considered of statistical significance.

## 3. Results

### 3.1. GC-MS Profiling of T. ericoides Bark Methanolic Extract

**Figure 1:**
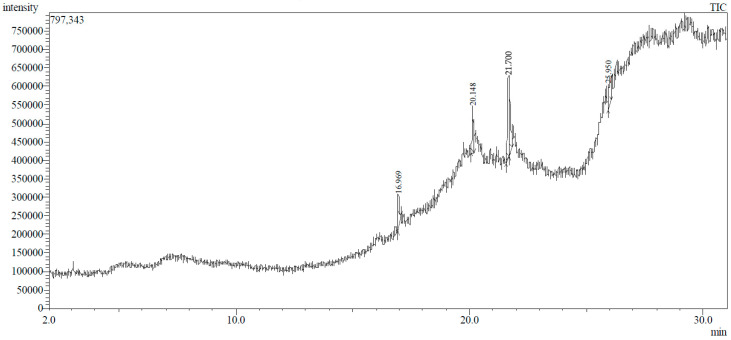
GC-MS chromatogram of methanolic *T. ericoides* bark extract.

The methanolic *T. ericoides* bark extract was analyzed for the presence of various compounds, and the identified phytocompounds from the methanolic fractions are presented in Figure 1. The identified compound structures were analyzed by mass fragmentation patterning and spectral data comparison with chemical profiles present in the National Institute of Standards and Technology (NIST) library. As seen in the figure, the chromatogram of distinct compounds, including diethyl phthalate, pentadecanoic acid, methyl 6,11-octadecadienoate, cyclopropaneoctanoic acid, 2-[(2-pentylcyclopropyl) methyl]-, methyl ester, and erythro-7,8-bromochlorodisparlure, were determined in the methanolic fractions of the *T. ericoides* bark extract. Also, the retention time, peak area, percentage peak area, height percentage, and names of the identified compounds are mentioned in Table 1.

### 3.2. Antibacterial Activity of T. ericoides Bark Methanolic Extract

**Figure 2:**
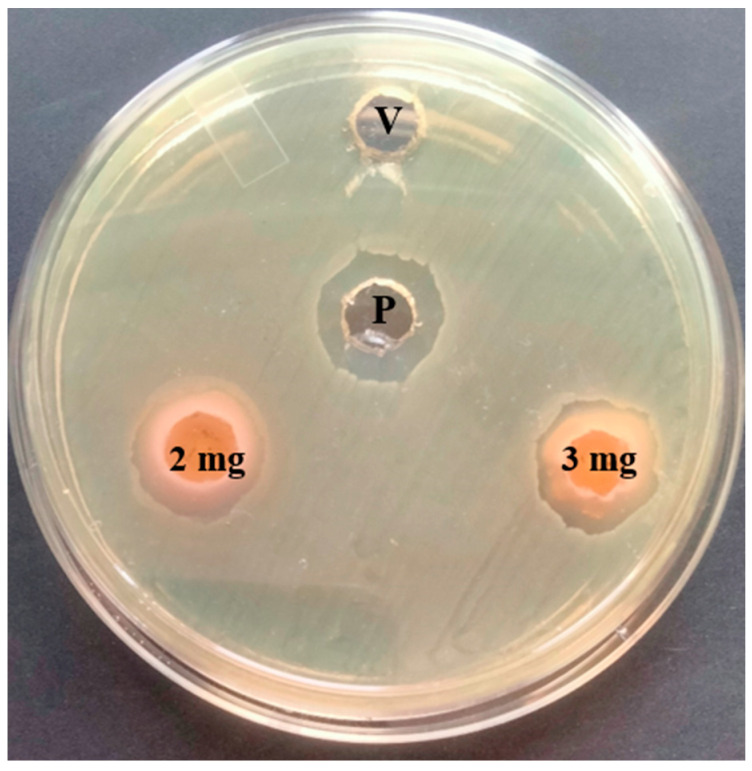
Antibacterial activity of *T. ericoides* bark methanolic extract against *E. faecalis.* Note: P—positive control and V—vehicle control.

The antibacterial activities of the *T. ericoides* bark methanolic extract against *E. faecalis* were investigated, and the observed result is presented in Figure 2. The inhibition of the growth of the *E. faecalis* is seen as a zone formation around the wells. It can also be noticed that higher concentrations of *T. ericoides* bark methanolic extract (2 mg and 3 mg/mL) increased the zone size, which indicates that the antibacterial activity of the *T. ericoides* bark extract against the *E. faecalis* was directly proportional to the extract concentration at this level.

### 3.3. MIC Determination for T. ericoides Bark Methanolic Extract

**Figure 3:**
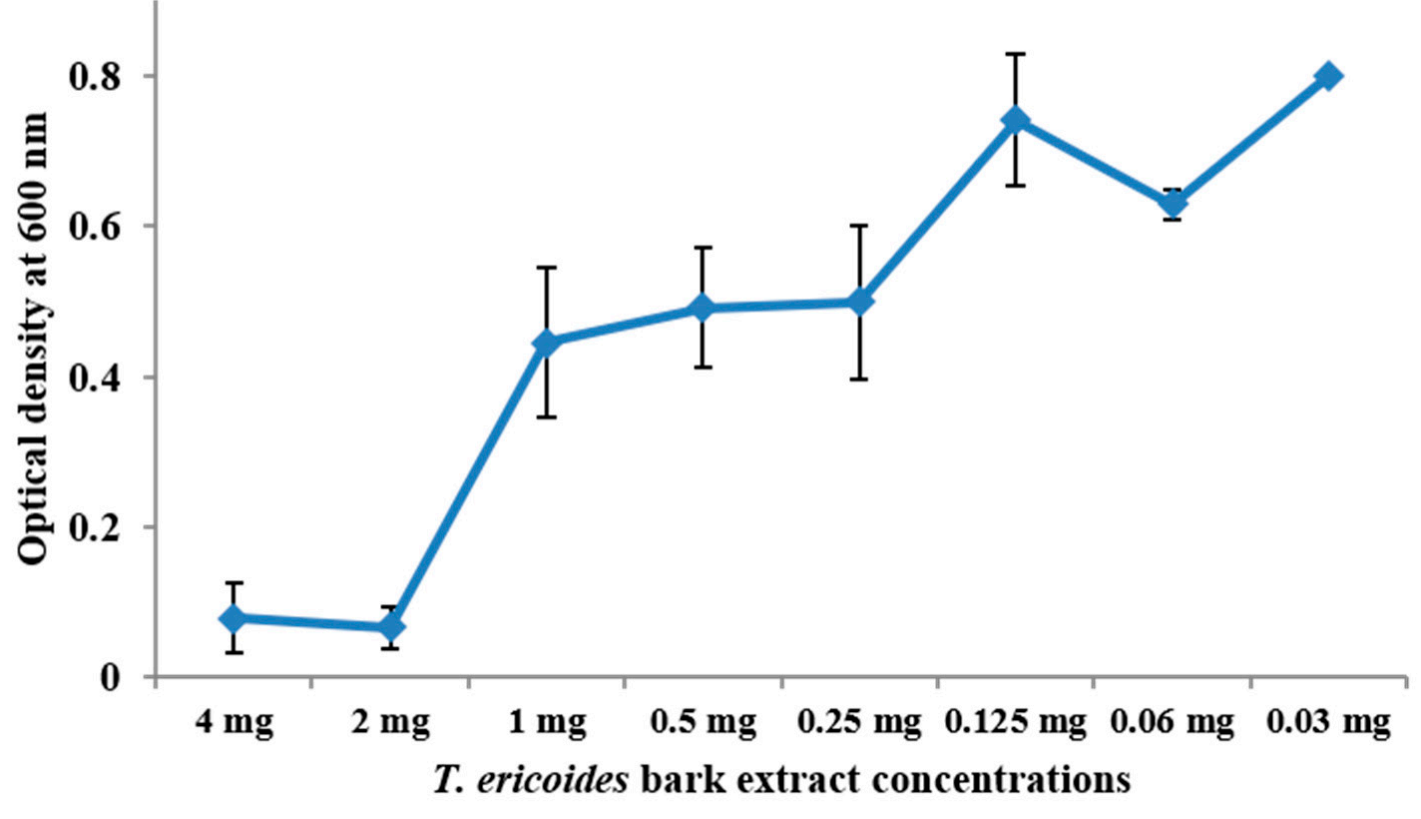
Graph showing the MIC of *T. ericoides* bark methanolic extract against *E. faecalis*.

The minimum concentration of *T. ericoides* bark methanolic extract needed to inhibit the growth of *E. faecalis* was calculated by the microdilution method, and the graph that was plotted against the *T. ericoides* bark methanolic extract against the measured optical density is presented in Figure 3. As seen in the figure, the MIC of the *T. ericoides* bark methanolic extract was found to be 2 mg/mL against the *E. faecalis.*

### 3.4. Time–Kill Assay of T. ericoides Bark Extract

**Figure 4:**
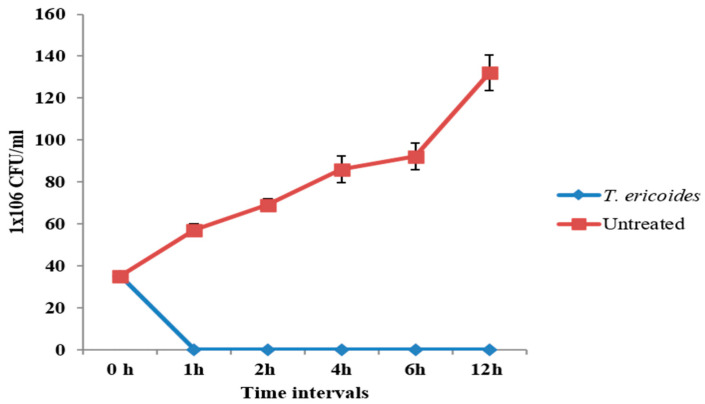
Killing kinetics *T. ericoides* bark extract against *E. faecalis* (noted at 1 h treatment).

The time required for the *T. ericoides* bark extract to kill the *E. faecalis* was calculated using time–kill assay, and the obtained result is displayed in Figure 4. As shown in the figure, when the *E. faecalis* was treated with 2 mg/mL of *T. ericoides* bark extract, it showed no viable cells after 1 h of treatment, which denotes the bactericidal effect of the *T. ericoides* bark extract, whereas more viable cells were observed in the untreated group.

### 3.5. Effect of T. ericoides Bark Extract on E. faecalis Biofilm Formation

**Figure 5:**
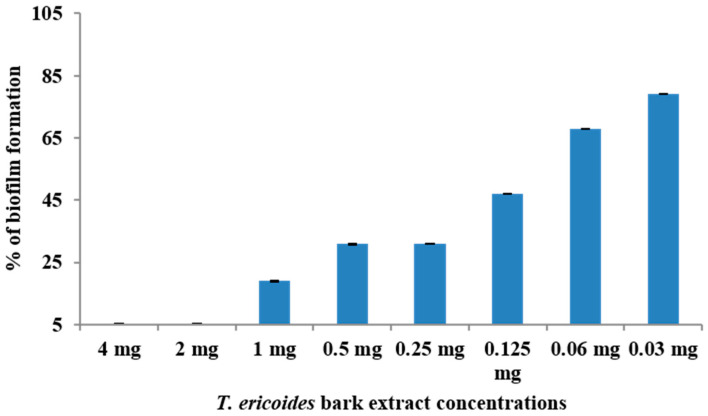
A graph showing the impact of *T. ericoides* bark extract on *E. faecalis* biofilm formation. The inhibition of biofilm was noted until the MIC (2 mg/mL).

The effect of the *T. ericoides* bark extract on biofilm formation by the *E. faecalis* was quantified through crystal violet assay, and the calculated percentages of the biofilm formation concerning various concentrations of *T. ericoides* bark extract are mentioned in Figure 5. The figure confirms the ability of *T. ericoides* bark extract to inhibit biofilm formation by the selected bacterial pathogen on polystyrene surfaces. Furthermore, it shows that the *T. ericoides* bark extract could inhibit the *E. faecalis* biofilm formation until its MIC, whereas slow biofilm formation was observed below the MIC, which represents that the biofilm formation of the *E. faecalis* was delayed with minimum concentrations of the extract.

### 3.6. Eradication Effect of T. ericoides Bark Extract on E. faecalis Biofilm

**Figure 6:**
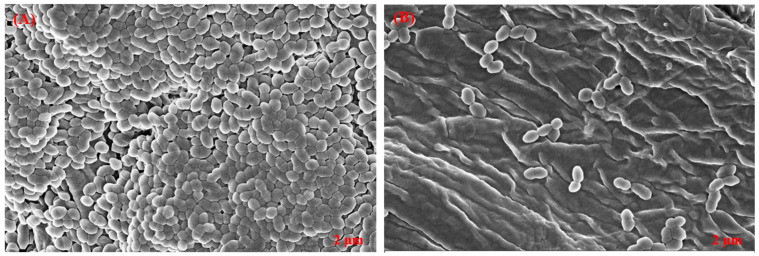
The effect of *T. ericoides* bark extract on the mature biofilms of *E. faecalis* was studied qualitatively using SEM. (**A**) Numerous viable cells adhered to the cellulose matrix. (**B**) 2 mg/mL of the *T. ericoides* bark extract treatment revealed a reduction in mature biofilms on the matrix. Scale bar—2 µm.

**Figure 7:**
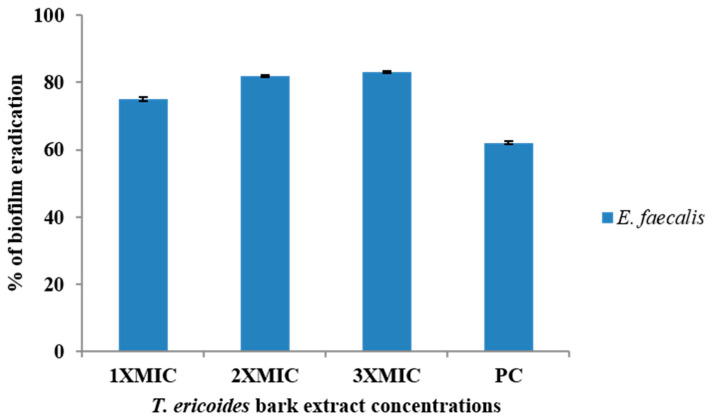
The effect of *T. ericoides* bark extract on the mature biofilms of *E. faecalis* was studied quantitatively using crystal violet assay, and the graph presents the percentage of biofilm eradication after treatments with three concentrations of *T. ericoides* bark extract. Note: PC—positive control.

The capability of the *T. ericoides* bark extract to eradicate the mature *E. faecalis* biofilms was investigated qualitatively on a cellulose matrix, and the obtained images which represent the biofilm reduction after the treatment are documented in Figure 6. The figure shows SEM images of many cells’ attachment on untreated cellulose matrices, whereas those treated with 2 mg/mL of *T. ericoides* bark extract showed reduced cell attachment. This indicates that *T. ericoides* bark extract has the ability to eradicate *E. faecalis* biofilms. Further, *E. faecalis* biofilm eradication after *T. ericoides* bark extract treatment with three different concentrations, viz., 1X MIC (2 mg/mL), 2X MIC (4 mg/mL), and 3X MIC (6 mg/mL), was quantified through crystal violet assay, and the result is shown in Figure 7. The graph represents the calculated percentages of biofilms eradicated after the treatments, which were 75%, 82%, and 83%, respectively.

### 3.7. Antibacterial Activity of T. ericoides Bark Extract-Coated Catheter

**Figure 8:**
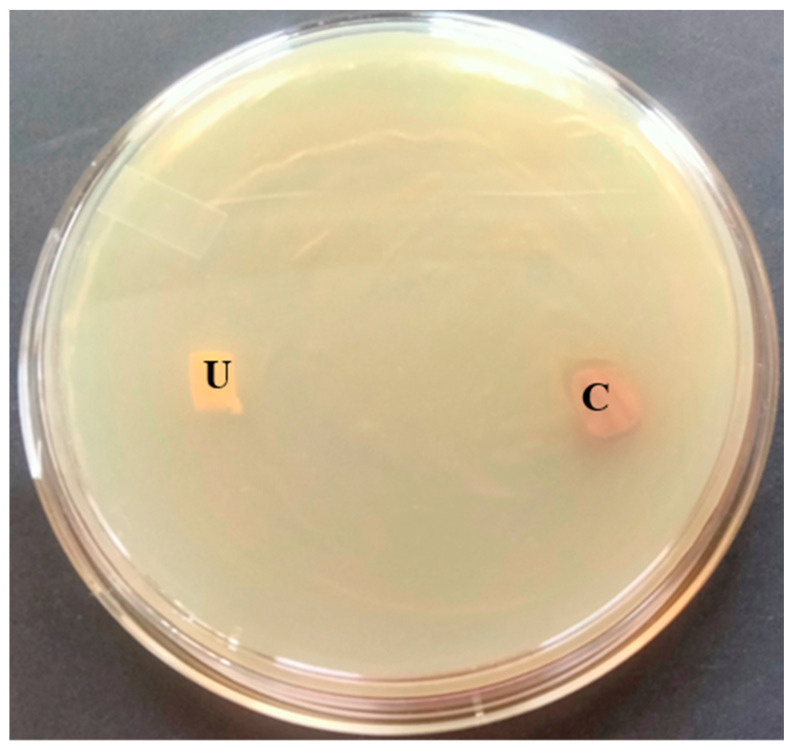
The antibacterial activity of the *T. ericoides* bark extract-coated catheter tube against the *E. faecalis* was investigated. The formation of a growth inhibition zone around the catheter coated with an extract tube against the *E. faecalis* is visible. Note: C—*T. ericoides* bark extract-coated tube; U—uncoated tube.

**Figure 9:**
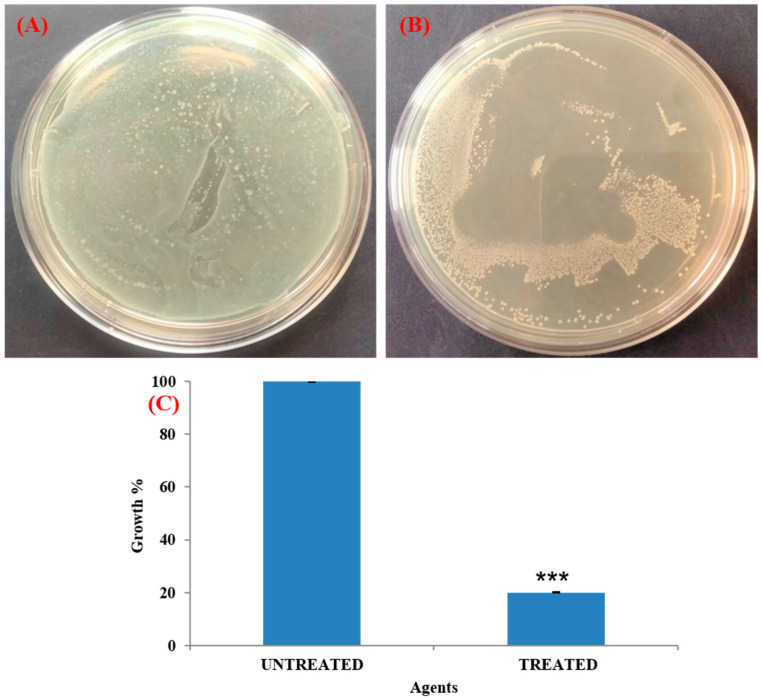
Quantification of *E. faecalis* load from *T. ericoides* bark extract-coated and uncoated catheter tube using viable count method. (**A**) Uncoated catheter tube produced greater number of CFUs. (**B**) Catheter coated with *T. ericoides* bark extract produced fewer CFUs. (**C**) Graph representing growth percentage calculated in catheter coated with *T. ericoides* bark extract. *** Highly significant.

The antibacterial activity of the *T. ericoides* bark extract-coated catheter tube against the *E. faecalis* was studied using an in vitro bladder model, and the observed growth inhibition around the catheter tube is represented in Figure 8. The diameter of the formed growth inhibitory zones determined the antibacterial activity of the extract against the *E. faecalis.* Further, the *T. ericoides* bark extract-coated catheter tube was analyzed for bacterial quantification through a viable cell count, and the percentage of growth that was calculated from the coated and uncoated catheters is presented in Figure 9. The figure shows more viable cells in the uncoated tube, whereas fewer viable cells were counted in the *T. ericoides* bark extract-coated catheter tube. Moreover, the growth percentage calculated after the extract treatment was 20% against the *E. faecalis*, which signified the anti-adhesive property of the *T. ericoides* bark extract-coated catheters.

### 3.8. Visualization of Biofilm on Bladder Model

**Figure 10:**
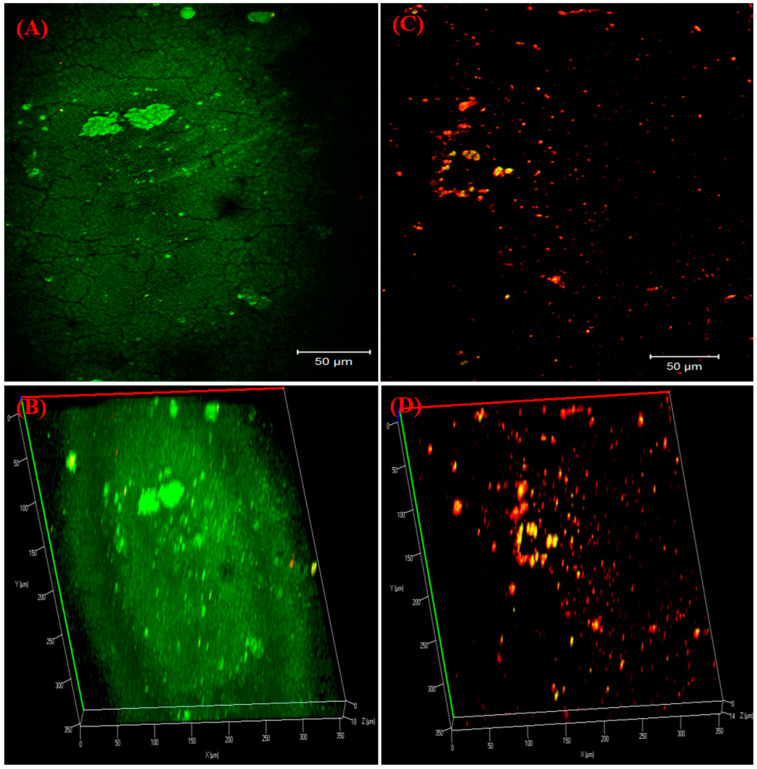
Biofilm visualization after staining with FDA and PI using CLSM (**A**) Visualization of biofilm formation on uncoated catheter, observed after 5 days of contact with *E. faecalis*. (**B**) Three-dimensional structure of biofilm formation on uncoated catheter surface, representing 18 µm thickness. (**C**) Visualization of biofilm formation on *T. ericoides* bark extract-coated catheter, observed after 5 days of contact with *E. faecalis*. (**D**) Three-dimensional view exposes reduction in biofilm thickness to 14 µm. Scale bar: 50 µm.

**Figure 11:**
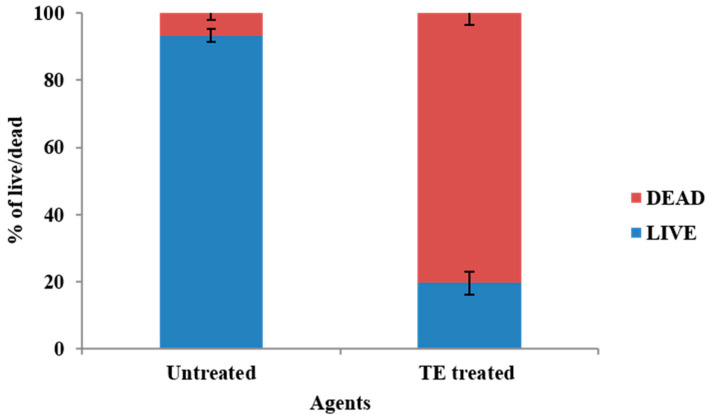
Based on the FDA and PI combination, the live and dead cell percentage was calculated from the uncoated and coated catheter tubes that were in contact with *E. faecalis* cells for 5 days and showed that 80% of cells were dead in the *T. ericoides* bark extract-coated catheter tube.

The anti-adhesive property of the *T. ericoides* bark extract-coated catheters against the *E. faecalis* was visualized using CLSM, and the live/dead percentage was calculated, as shown in Figure 10A–D. Figure 10A represents the uncoated catheter tube which was allowed to come into contact with *E. faecalis* cells for 5 days and stained with fluorescein diacetate (FDA, emits green fluorescence) which binds to live cells and propidium iodide (PI, emits red fluorescence) which binds to DNA present in membrane-damaged cells. The three-dimensional *E. faecalis* biofilm structure noted with an 18 µm thickness on the uncoated catheter tube is shown in Figure 10B. As visualized in Figure 10C, the *T. ericoides* bark extract-coated catheter tube emitted a high range of red fluorescence which represents that the extract caused membrane damage; thereby, PI entered into the cells and bound to the DNA. The anti-adhesive property of the *T. ericoides* bark extract against the *E. faecalis* was explored on the extract-coated catheter tube, resulting in a reduced three-dimensional biofilm thickness (14 µm). Further, based on the FDA and PI combination, the calculated live/dead percentage is presented in Figure 11, which reveals that 80% of cells were dead on the *T. ericoides* bark extract-coated catheter.

### 3.9. Impact of T. ericoides Bark Extract on E. faecalis Morphology

**Figure 12:**
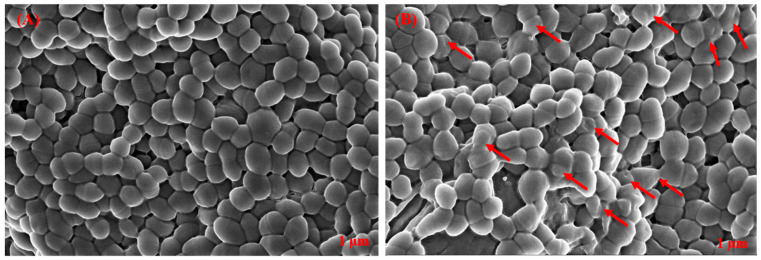
The effect of *T. ericoides* bark extract on *E. faecalis* cell morphology (**A**) *E. faecalis* cells without treatment showed smooth and undamaged cell surfaces. (**B**) Red arrows point *E. faecalis* cells which showed ballooning and cell shrinkage after treatment with *T. ericoides* bark extract.

The effect of the *T. ericoides* bark extract on the *E. faecalis* cell morphology was studied using SEM, and the images obtained are presented in Figure 12. As seen in the figure, the *E. faecalis* cells treated with 2 mg/mL of *T. ericoides* bark extract for 1 h induced internal component leakage, resulting in cell shrinkage, which was compared to the untreated *E. faecalis* cells, where undamaged and smooth cell surfaces were noted.

### 3.10. Cytotoxicity of T. ericoides Bark Extract

**Figure 13:**
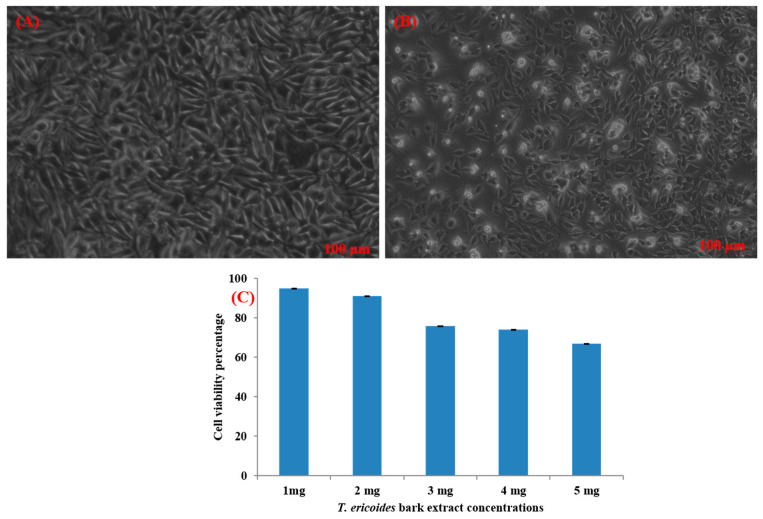
Cytotoxic effect of *T. ericoides* bark extract on L_929_ cells. (**A**) Untreated L_929_ cells. (**B**) L_929_ cells treated with *T. ericoides* bark extract. (**C**) Graph representing the cell viability percentage after various concentrations.

The cytotoxicity of the *T. ericoides* bark extract was investigated against the L_929_ cells, and the results are displayed in Figure 13. The graph denotes the L_929_ cell viability as 95%, 91%, 76%, 74%, and 67% after 1 mg/mL, 2 mg/mL, 3 mg/mL, 4 mg/mL, and 5 mg/mL of *T. ericoides* bark extract treatment, respectively, which was compared to the untreated cells.

## 4. Discussion

Nosocomial infections associated with medical devices are of great concern in patients hospitalized for long periods of time. CAUTI is one of the most important nosocomial infections, and when it is caused by virulent, biofilm-forming bacteria like *E. faecalis*, its treatment and management become challenging. Along with other reasons like the overuse of antibiotics, biofilm formation also leads to the development of antibiotic-resistant strains. This alarming condition has provoked the finding of new antibacterial agents of natural origin to fight against CAUTI-causing organisms. Hence, our study explored the antibacterial, antibiofilm activities of the bark of a less-explored medicinal plant, *T. ericoides*, and its methanolic extract was screened against *E. faecalis*, one of the major biofilm pathogens involved in CAUTIs. This investigation found that the bark extract possessed potent antibacterial activity, with its very lowest concentrations being as low as 2 mg/mL, against *E. faecalis*.

The present study was supported by an earlier work in which the antimicrobial activities of an extract of *T. ericoides* were studied using different solvents like methanol, ethanol, aqueous solutions, and petroleum ether against *Bacillus subtilis*, *Salmonella typhi*, *Escherichia coli*, and *Candida albicans.* Among them, the methanol extract showed potent antimicrobial activity against the pathogens, which was compared with other solvents [48]. In the same way, a recent study has been reported for another species of plant from the same family, *T*. *nilotica* (Ehrenb) Bunge, and its n-butanol fractions showed potent antibacterial activity against *Klebsiella pneumoniae* [49]. *T. aphylla*, a medicinal plant from Saudi Arabia, is the most studied species from the Tamaricaceae family and was investigated for its antimicrobial potentials against *E. coli*, *Bacillus subtilis*, *Salmonella typhi*, *Staphylococcus aureus*, *Aspergillus flavus*, and *C. albicans*. The results showed that both the methanolic and ethanolic leaf extracts of the plant possessed promising activities against all of the selected microbial pathogens with no differences in their MICs against the test pathogens [50,51,52]. Other species from the Tamaricaceae family like *T. gallica* and *T. ramosissima* were also explored for their antimicrobial activities against Gram-negative bacterial pathogens, and both plants displayed promising antimicrobial activities due to the presence of phytochemicals in the extracts [53,54,55,56]. Overall, the studies suggested that antimicrobial activity is not solely dependent upon the solvents used for their extractions. In the present study, the antibacterial activities of the bark extract were further confirmed by investigating their killing ability against *E. faecalis* and displayed novel bactericidal effects.

The present study explored the presence of various phytochemicals, including diethyl phthalate, pentadecanoic acid, methyl 6,11-octadecadienoate, cyclopropaneoctanoic acid, 2-[(2-pentylcyclopropyl) methyl]-, methyl ester, and erythro-7,8-bromochlorodisparlure in the methanolic fractions of the *T. ericoides* bark extract, which are responsible for antibacterial activity against *E. faecalis.* The phytochemical profiling of *T. aphylla* hexane fractions showed some major compounds like 6,10,14-trimethyl-2-pentadecanone, dodecanoic acid, and octadecane along with some minor compounds [57]. *T. ramosissima* bark extract has also been reported to possess hispidulin, isorhamnetin, and cirsimaritin, which can inhibit the formation of 2-amino-1-methyl-6-phenylimidazo [4,5-*b*] pyridine and thus represents excessive beneficial effects on human health [58]. Various studies on the antibacterial activities of phytochemicals, such as diethyl phthalate, pentadecanoic acid, methyl 6,11-octadecadienoate, cyclopropaneoctanoic acid, 2-[(2-pentylcyclopropyl) methyl]-, methyl ester, erythro-7,8-bromochlorodisparlure, etc., against many human pathogens like *S. aureus*, *Pseudomonas aeruginosa*, *C. albicans*, and *Listeria monocytogenes* have shown potent antimicrobial activity [59,60,61,62]. Thus, the above findings indicate that each species has different phytochemicals with various applications.

The antibiofilm activities of the *T. ericoides* bark extract against *E. faecalis*, a biofilm-forming uropathogen that enters through the lumen space and becomes attached to catheter surfaces, colonizing and forming matured biofilm and making treatment ineffective [63], was also analyzed. This study focused on every stage of biofilm formation to avert the biofilms during the formation process itself. The *T. ericoides* bark extract was able to inhibit biofilm formation on polystyrene surfaces until its MIC level was reached and it was quantified and again confirmed through SEM, and the biofilm reduction was visualized after the treatment. In addition, the effect of the *T. ericoides* bark extract on the *E. faecalis* mature biofilms was also quantified, and, hence, the antibiofilm activity was proved. The present study was supported by a recent report wherein *T. nilotica* dichloromethane (DCM) and ethyl acetate (EtOAc) fractions were evaluated for their antifungal activities against *C. albicans*; their potential actions were reported, with MICs ranging from 64 to 256 and 128 to 1024 μg/mL, respectively. Further, the DCM fraction of *T. nilotica* reduced the biofilm formation, and it was evidenced by SEM analysis, indicating that *T. nilotica* might be an important antifungal agent against *C. albicans* [64]. Another study reported that developed silver- and silver-polytetrafluoroethylene combined nanocomposite-coated catheters exhibited potent antibacterial and antiadhesive activities against two important CAUTI-causing organisms: *E. coli* WT F1693 and *S. aureus* F1557. The catheters coated with silver and with Ag-PTFE composite were able to reduce bacterial adhesion by up to 60.3% and 55.2%, respectively. However, the present study significantly reduced the biofilm adhesion by up to 80% when compared to the uncoated catheters [65].

Apart from protecting against *E. faecalis* via defense mechanisms and antibiotic treatment, biofilm formation on the surface of urinary catheters may also affect catheter function [66]; hence, coating the catheters with any antimicrobials is an excellent method to avoid biofilms on the outer and the inner surface of the catheters [67]. Therefore, this study examined the coating of the catheter surface with *T. ericoides* bark extract and its antibacterial activity against *E. faecalis*. It found potent activity against the test pathogen, and the activity was further quantified using the viable cell count method which suggested that catheter coating with the extract could prevent biofilm formation on the surface of the catheter. Further, to visualize the biofilm inhibition on the catheter surface, FDA and PI staining were used to differentiate the biofilm formation on the extract-coated and uncoated catheter surfaces. By analyzing the staining patterns, structured biofilm formation was noted on the uncoated catheter and reduced biofilm formation was observed on the extract-coated catheter surface, which indicated that the *T. ericoides* bark extract-coated catheter efficiently prevented biofilm formation on the catheter surface. Here, the *T. ericoides* bark extract was able to damage the bacterial cell membrane upon treatment, resulting in PI entering into the membrane and binding to the DNA, thereby enabling the observation of red fluorescence. In support of this, numerous reports have documented that antimicrobials, such as antibiotic combinations, fosfomycin, silver nanoparticles, polymer, zinc oxide coatings, etc., on catheter tubes have shown excellent antimicrobial activity against *S. aureus*, *E. faecalis*, *K. pneumoniae*, and *E. coli* [68,69,70].

The present investigation showed that the morphology of the *E. faecalis* cells was damaged when treated with the *T. ericoides* bark extract, resulting in osmotically unbalanced cells which created permeabilization leading to the leakage of intracellular components, resulting in cell death, and this was evident in the SEM analysis. This suggests a possible mechanism of action of the *T. ericoides* bark extract against *E. faecalis*; this could be the membrane damage which was already confirmed in the CLSM analysis. The aim of this study was envisioned for human use; therefore, the toxicity of the *T. ericoides* bark extract was tested for safety. The results showed that the extract was nontoxic. Even though the bark extract coating of the catheters reduced the infection by interfering with the biofilm formation of the bacterial pathogen in vitro, our study must overcome the current limitation by conducting an in vivo animal model to prove the antibiofilm and anti-adhesive activities of the extract against *E. faecalis*. So, further studies should be performed to understand the practicability and efficiency of the extract as a catheter-coating agent to prevent biofilm formation by the selected bacterial pathogen. Thus, while establishing the antibiofilm and antiadhesive activities of the methanolic bark extract of *T. ericoides* against *E. faecalis*, the present study recommends further, detailed, in vivo studies to make a potential catheter-coating agent.

## 5. Conclusions

Catheter-associated urinary tract infections (CAUTIs), when caused by biofilm-forming bacterial pathogens like *E. faecalis*, pose a real challenge by making the treatment and the management of the infection complicated. As the scientific world is searching for novel antibiofilm agents such as catheter coatings, the present study tried to explore the antibiofilm and antiadhesive potentials of a methanolic extract of the bark of one of the least exploited medicinal plants, *T. ericoides*, against *E. faecalis*, an important CAUTI-causing uropathogen. The extract displayed promising antibacterial activity at low concentrations owing to the presence of phytochemicals in methanolic fractions. The killing ability of the *T. ericoides* bark methanolic extract was calculated and the antibiofilm activity was verified based on the biofilm inhibition and mature biofilm eradication. The in vitro bladder model provided excellent anti-adhesive activity against the *E. faecalis* as the extract-coated catheter could reduce the bacterial adhesion by 80% when compared to the uncoated silicone catheters, and this was confirmed through CLSM. Based on the above findings, the authors recommend that the bark extract of *T. ericoides* can be developed as a potential catheter-coating agent after detailed studies.

## Figures and Tables

**Table 1:** List of phytochemicals present in methanolic fractions of *T. ericoides* bark extracts through GC-MS.

Peak No.	Retention Time	Peak Area %	Height %	Compound Name	Structure
1	16.969	12.42	11.82	Diethyl phthalate	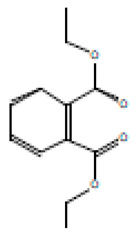
2	20.148	14.53	17.73	Pentadecanoic acid	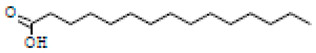
3	21.670	24.91	31.24	Methyl 6,11-octadecadienoate	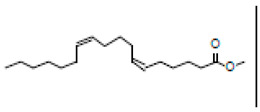
4	21.700	33.16	29.54	Cyclopropaneoctanoic acid, 2-[(2-pentylcyclopropyl)methyl]-methyl ester, trans	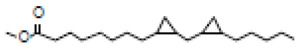
5	25.950	14.98	9.67	Erythro-7,8-bromochlorodisparlure	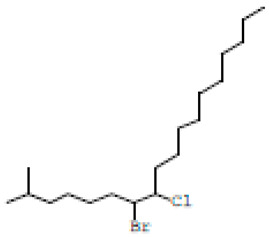

## Data Availability

No new data were created or analyzed in this study. Data sharing is not applicable to this article.
